# Identification of a Hydrogen-Sulfide-Releasing Isochroman-4-One Hybrid as a Cardioprotective Candidate for the Treatment of Cardiac Hypertrophy

**DOI:** 10.3390/molecules27134114

**Published:** 2022-06-27

**Authors:** Yu Wang, Yuechen Liu, Hongyu Wu, Shengtao Xu, Fenfen Ma

**Affiliations:** 1 Key Laboratory of Cardiovascular and Cerebrovascular Medicine, Nanjing Medical University, Nanjing 211166, China; yuwang06080028@163.com (Y.W.); liuyuechen9729@163.com (Y.L.); 2 State Key Laboratory of Natural Medicines, Department of Medicinal Chemistry, China Pharmaceutical University, 24 Tong Jia Xiang, Nanjing 210009, China; wuhyu0504@163.com; 3 Department of Pharmacy, Shanghai Pudong Hospital, Fudan Univerisity, Shanghai 201399, China; 4 School of Pharmacy, Fudan University, Shanghai 201203, China

**Keywords:** H_2_S donors, cardiac hypertrophy, cardiac fibrosis, AMPK, isochroman-4-one, hybrids

## Abstract

Cardiac pathological hypertrophy is associated with undesirable epigenetic changes and causes maladaptive cardiac remodeling and heart failure, leading to high mortality rates. Specific drugs for the treatment of cardiac hypertrophy are still in urgent need. In the present study, a hydrogen-sulfide-releasing hybrid **13-E** was designed and synthesized by appending *p*-hydroxythiobenzamide (TBZ), an H_2_S-releasing donor, to an analog of our previously discovered cardioprotective natural product XJP, 7,8-dihydroxy-3-methyl-isochromanone-4. This hybrid **13-E** exhibited excellent H_2_S-generating ability and low cellular toxicity. The **13-E** protected against cardiomyocyte hypertrophy In Vitro and reduced the induction of *Anp* and *Bnp*. More importantly, **13-E** could reduce TAC-induced cardiac hypertrophy In Vivo, alleviate cardiac interstitial fibrosis and restore cardiac function. Unbiased transcriptomic analysis showed that **13-E** regulated the AMPK signaling pathway and influenced fatty acid metabolic processes, which may be attributed to its cardioprotective activities.

## 1. Introduction

Cardiac hypertrophy is usually characterized by an increase in cardiomyocyte size and the thickening of ventricular walls. Cardiac hypertrophy is commonly classified as physiological, when it is associated with normal cardiac function, or as pathological, when it is associated with cardiac dysfunction [1]. Pathological hypertrophy is a risk factor which is induced by factors such as prolonged and abnormal hemodynamic stress, including hypertension, myocardial infarction, etc. [2]. Pathological hypertrophy is associated with fibrosis, capillary rarefaction, increased production of pro-inflammatory cytokines, cellular dysfunction and undesirable epigenetic changes, leading to maladaptive cardiac remodeling and heart failure [3]. Until now, although many cardioprotective drugs have been found to alleviate hypertrophy, such as diuretics, angiotensin converting enzyme inhibitors (ACEI), angiotensin II receptor blockers and beta-blockers, mortality rate remains high, and specific drugs for the treatment of cardiac hypertrophy are still in urgent need [4].

**Figure 1:**
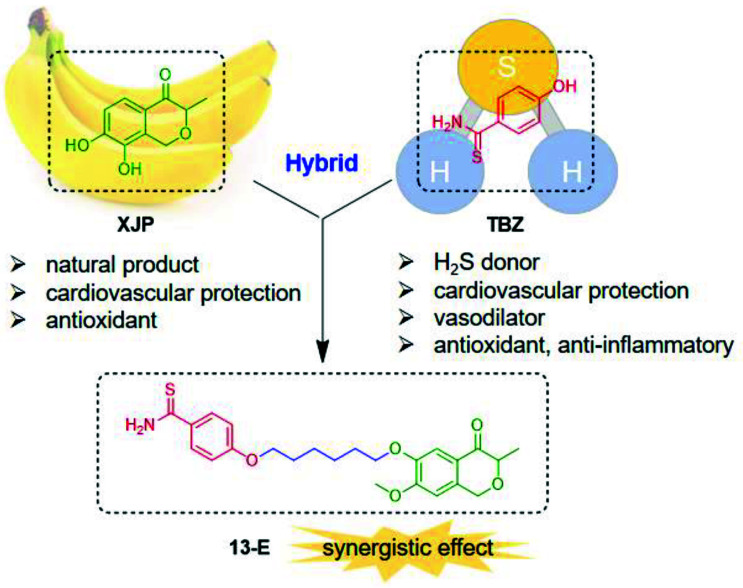
Design strategy of hydrogen-sulfide-releasing XJP hybrid **13-E**.

The discovery of active molecules from natural products is an important strategy to develop novel cardioprotective drugs. Many bioactive derivatives from nature, including polyphenolic compounds, peptides, oligosaccharides, vitamins and unsaturated fatty acids, possess protective effects on cardiovascular diseases [5,6]. 7,8-dihydroxy-3-methyl-isochromanone-4 (XJP, Figure 1) is a structurally unique natural polyphenolic compound, isolated from the banana (*Musasapientum* L.) peel by our group previously. Our biological evaluations showed that XJP possesses a variety of biological activities, including antiinflammation [7], antihypertensive [8,9], antioxidative activities [10] and even the prevention of Alzheimer’s disease [11]. It has been reported that XJP has excellent antihypertensive activity in spontaneously antihypertensive rats (SHRs) [8]. In addition, XJP was found to inhibit the ox-LDL-induced endothelial dysfunction, partly due to its anti-oxidant activity and its ability to modulate the PI3K/Akt/eNOS signaling pathway [10]. Importantly, XJP was identified as a novel ACEI and exhibits inhibitory activity on lipopolysaccharide (LPS)-accelerated vascular inflammation [7]. Based on our previous studies, we are therefore proposing that XJP may alleviate cardiac hypertrophy, which has not been studied in previous work.

Hydrogen sulfide (H_2_S) is an endogenous gas signaling molecule, which was first believed to be a toxic byproduct of metabolic processes until Kimura demonstrated H_2_S as an endogenous neuromodulator and discovered its physiological role in regulating smooth muscle relaxation [12]. H_2_S is now regarded as the third gasotransmitter besides nitric oxide (NO) and carbon monoxide (CO) [13]. Mounting evidence has indicated the wide range of physiological and pathological activities of H_2_S in cardiovascular systems endowed with antioxidant, anti-inflammatory, pro-autophagic and cardioprotective properties [14]. Although H_2_S plays an important role in cardiac pathology and physiology, it is not suitable for direct use in clinical treatment due to uncontrollable doses and high toxicity. The use of H_2_S donors represents an exciting and intriguing strategy to be pursued for the treatment of cardiovascular disease [15]. In recent years, H_2_S-releasing compounds, such as GYY4137, have been developed and show promising cardioprotective activities. For example, Xie et al. found that GYY4137 can alleviate atherosclerosis through increasing the expression of HO-1, and Meng et al. found that GYY4137 protects the heart from hypertrophy [16,17]. Our group also developed novel allyl thioesters as potential cardioprotective agents by releasing H_2_S [18].

In recent years, several H_2_S-releasing natural product hybrids have been developed by using the combination principle, in which a H_2_S-releasing moiety was linked to a natural product, to improve activity or reduce side effects. These hybrids may exhibit greater activities than their respective parent natural product, but with less toxicity, representing a superior design strategy for developing H_2_S donors [19,20]. Given the pharmacological effects of XJP, including its antihypertensive and cardioprotective activities, it is closely related to the etiology of cardiac hypertrophy, and there has been extensively reported cardioprotective activity of H_2_S for cardiac hypertrophy. Our design principle proposes a method to fuse the important pharmacophore of XJP and H_2_S donors into a new molecule to further improve its properties and to enhance its activity. (Figure 1).

In the present study, a more potent and stable analog of XJP, by protecting the phenolic hydroxyl group, was selected as the parent natural product [21], and *p*- hydroxythiobenzamide (TBZ) was used as the H_2_S-releasing moiety, as it is a widely used H_2_S-releasing compound characterized with controllable and slow-releasing properties [22]. The target hybrid **13-E** was firstly designed and synthesized by linking the TBZ group to XJP by a flexible alkyl link, and its effects towards hypertrophy induced by transverse aortic constriction (TAC) were explored. The protective effects of **13-E** were tested using primary cultured cardiomyocytes in addition to a well-established animal model of cardiac hypertrophy. The results showed that **13-E** possesses good cardioprotective activities and attenuates hypertrophy potently, which deserves further investigations.

## 2. Results

### 2.1. Synthesis and Characterization of Hybrid **13-E**

**Scheme 1:**
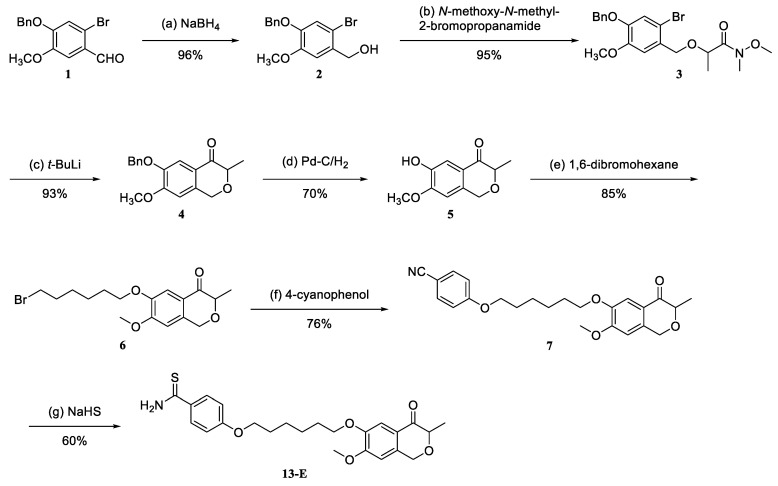
The synthetic route of target compound **13-E**.

The synthesis and structure identification of XJP has been reported by our group previously [23]. Briefly, the commercially available benzaldehyde **1** was reduced to alcohol **2** with sodium borohydride in an almost quantitative yield. The subsequent reaction of alcohol **2** with *N*-methoxy-*N*-methyl-2-bromopropanamide afforded Weberamide **3** in a 95% yield. Isochroman-4-one **5** was obtained by the cyclization of **3** with *t*-BuLi as a base, following the deprotection of benzyl in the presence of Pd-C/H_2_. Finally, compound **5** engaged in nucleophile substitution with 1,6-dibromohexane to afford compound **6**, which then reacted with 4-cyanophenol to produce intermediate **7**. The reaction of NaHS and intermediate **7** with appropriate amounts of MgCl_2_·6H_2_O in the solvent of DMF gave the target compound **13-E** in a 60% yield (Scheme 1). The structure of compound **13-E** was identified unambiguously with ^1^H-NMR, ^13^C-NMR and HR-MS (please see Supporting Information).

*Reagents and conditions:* (a) NaBH_4_, MeOH, rt, 30 min; (b) *N*-methoxy-*N*-methyl-2-bromopropanamide, NaH, rt, dry DMF, 30 min; (c) *t*-BuLi, −78 °C, 1 min, then H_2_O; (d) Pd-C/H_2_, THF, rt, 4 h; (e) 1,6-dibromohexane, K_2_CO_3_, dry acetone, reflux, 2 h; (f) 4-cyanophenol, K_2_CO_3_, dry acetone, reflux, 6 h; (g) NaHS, MgCl_2_·6H_2_O, DMF, 12 h.

### 2.2. H_2_S-Releasing Capability and Safety of **13-E**

**Figure 2:**
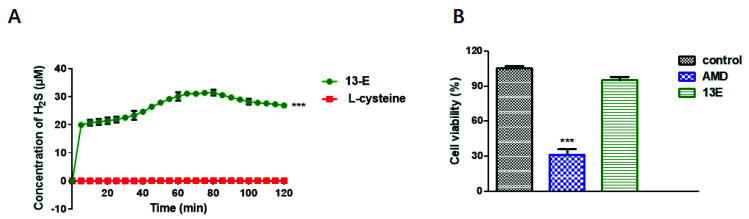
H_2_S-releasing capability and safety of **13-E**: (**A**) H_2_S-releasing ability of compound **13-E** (100 μM), values are expressed as mean ± SD, *n* = 3; (**B**) Effects of compound **13-E** and positive control AMD on cell viability of HUVEC cells. Statistical significance was analyzed using ANOVA, and values are expressed as mean ± SEM, *n* = 3; *** *p* < 0.001 as compared with the negative control.

We first tested the H_2_S-releasing capability of **13-E** by using the methylene blue (MB) method. As shown in Figure 2A, **13-E** exhibited excellent H_2_S-generating ability and produced H_2_S rapidly with a peak time of around 5–10 min, whereas the negative control L-cysteine did not release H_2_S. It is interesting that **13-E** released H_2_S smoothly for a period of time with a maximum of 31.9 μM of H_2_S at around 1 h and only showed a slight downward trend since 80 min, which is consistent with the slow-releasing process of H_2_S In Vivo. The effects of compound **13-E** on cell viability were then evaluated to investigate the safety of **13-E**, and adriamycin (AMD) was used as a positive control. As expected, **13-E** at a concentration of 100 μM had no effects on cell viability, indicating the safety of this compound (Figure 2B).

### 2.3. **13-E** Protects against Cardiomyocyte Hypertrophy In Vitro

**Figure 3:**
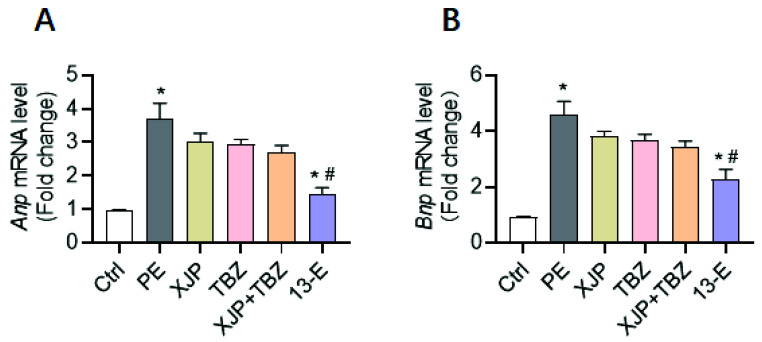
PE-induced cardiomyocyte hypertrophy markers were significantly increased, whereas mRNA level of (**A**) *Anp* and (**B**) *Bnp* could be mitigated by co-incubation with **13-E**; *n* = 3 independent experiments. The results are presented as mean ± SEM. * *p* < 0.05, versus ctrl; # *p* < 0.05, versus PE.

We further determined the impact of **13-E** on the development of cardiac hypertrophy In Vitro. In order to determine whether the anti-hypertrophic efficiency of **13-E** was better than the XJP or combination of the XJP with TBZ, we isolated neonatal rat ventricular cardiomyocytes (NRVCs) and incubated the cells with XJP, TBZ, XJP+TBZ and **13-E** and detected the induction of *Anp* and *Bnp* after phenylephrine (PE) treatment. The results show that the level of XJP (10 μM), TBZ (10 μM) or XJP (10 μM)+TBZ (10 μM) on *Anp* and *Bnp* after PE treatment had no statistical difference. In contrast, **13-E** significantly reduced the induction of *Anp* and *Bnp* by PE, suggesting an antihypertrophic role of **13-E** (Figure 3).

### 2.4. **13-E** Restores Cardiac Function

**Figure 4:**
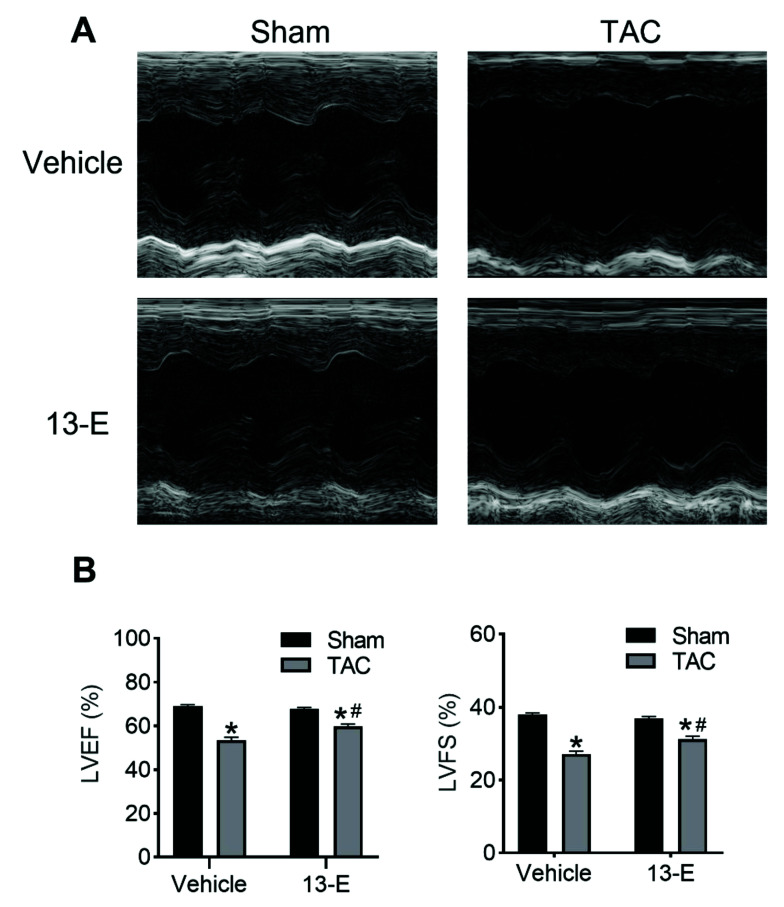
Eight-week-old mice were treated with vehicle or 20 mg/kg **13-E** (i.p.), which were performed with sham or TAC operation for four weeks (*n* = 8 for each group): (**A**) Representative echocardiographic of cardiac section for each condition; (**B**) Bar graphs showing quantitative data for LVEF and LVFS. The results are presented as mean ± SEM. * *p* < 0.05, versus sham; # *p* < 0.05, versus vehicle + TAC. Comparisons across all groups were performed by two-way ANOVA with Tukey’s post hoc multiple comparisons.

To further demonstrate the cardioprotective effects of **13-E** In Vivo, sham or TAC mice were subjected to the vehicle or 20 mg/kg **13-E** (i.p.) for 4 weeks. There was no difference in the heart beats (BMP) of mice between the sham and TAC groups (Figure S1). The echocardiographic results show that TAC groups distinctly decreased the functional parameters of the left ventricular ejection fraction (LVEF, %) and left ventricular fractional shortening (LVFS, %) as compared to the sham group, and these effects could be alleviated by **13-E** (Figure 4). Additionally, both the left ventricular end-diastolic volume (LV vol; d, μL) and left ventricular end-systolic volume (LV vol; s, μL) were remarkably increased in vehicle mice subjected to TAC, which were reversed in **13-E**-treated mice (Figure S2A,B). These results manifest that **13-E** may contribute to cardiac functions.

### 2.5. **13-E** Reduces TAC-Induced Cardiac Hypertrophy

**Figure 5:**
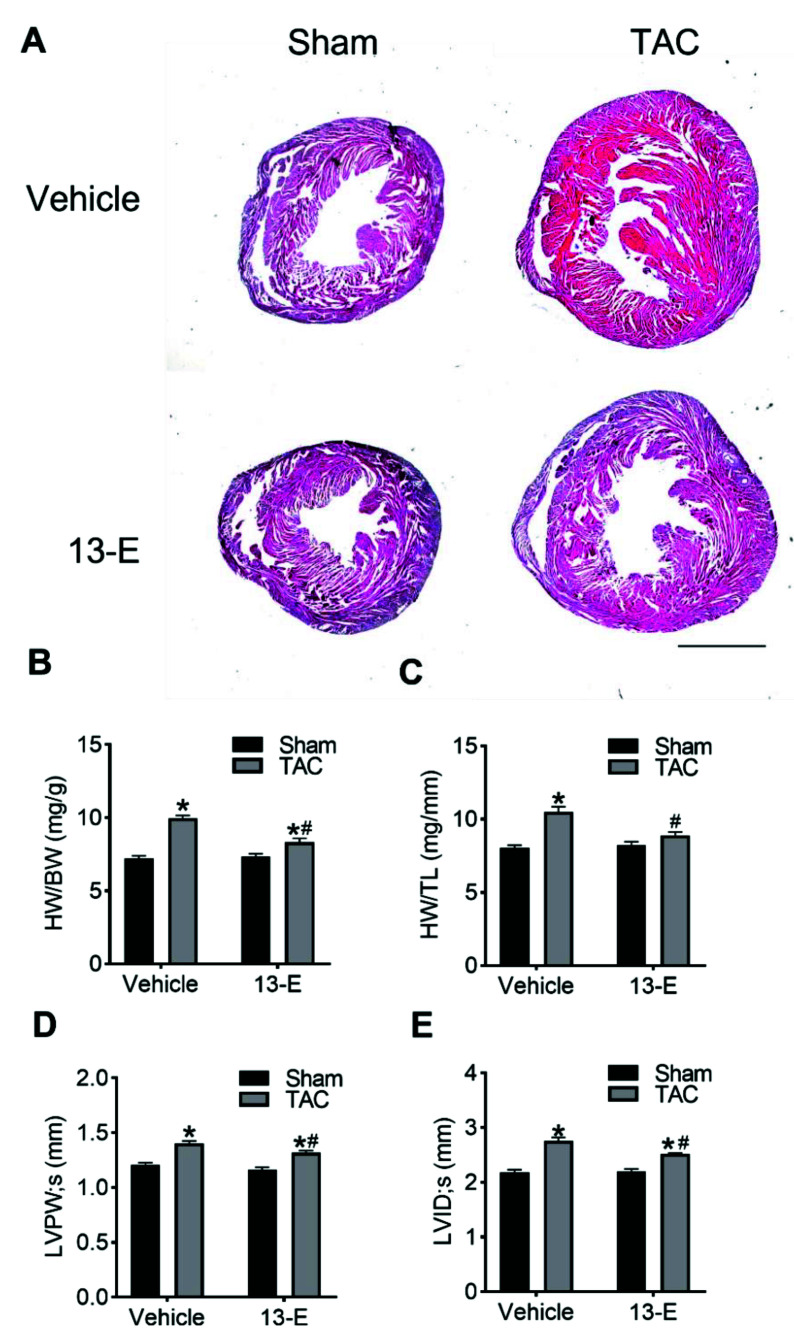
Eight-week-old mice were treated with vehicle or 20 mg/kg **13-E** (i.p.), which were performed with sham or TAC operation for four weeks (*n* = 8 for each group): (**A**) Representative H and E staining (Scale Bars: 1 mm) graphs of cardiac sections from mice treated with vehicle or **13-E**. (**B**) Ratios of heart weight to body weight (HW/BW, mg/g); (**C**) Heart weight to tibia length (HW/TL, mg/mm) of hearts from the sham + vehicle, TAC + vehicle, sham + **13-E** and TAC + **13-E** groups were determined; (**D**) LVPW in end-systolic dimensions (s, mm) and (**E**) LVID (s, mm) values were analyzed in vehicle or **13-E** treatment in sham- or TAC-operated mice. The results are presented as mean ± SEM. * *p* < 0.05, versus sham; # *p* < 0.05, versus vehicle + TAC. Comparisons across all groups were performed by two-way ANOVA with Tukey’s post hoc multiple comparisons.

The TAC model displayed the development of left ventricular hypertrophy and wall thickening and the progressive development of left ventricular systolic and diastolic dysfunction. Hematoxylin and Eosin (H and E) staining was further performed to evaluate the histological features of cardiac hypertrophy. As shown in Figure 5A, **13-E** treatment restrained the increase in heart size caused by TAC surgery. Furthermore, TAC-induced hypertrophy manifested the increase in ratios of heart weight to body weight (HW/BW, mg/g) or tibia length (HW/TL, mg/mm) compared to mice in the sham group. This consequence was availably repressed by **13-E** administration (Figure 5B,C). The cardiac-hypertrophy-related parameters, such as the left ventricular posterior wall dimension (LVPW, mm), the left ventricular internal dimension (LVID, mm) and the interventricular septum thickness (IVS, mm), were increased in the TAC group and were later suppressed by **13-E** administration (Figure 5D,E; Figure S2C–F). These results demonstrate that **13-E** is capable of preserving left ventricular functions and delaying the progression of cardiac hypertrophy.

### 2.6. **13-E** Alleviates Cardiac Interstitial Fibrosis

**Figure 6:**
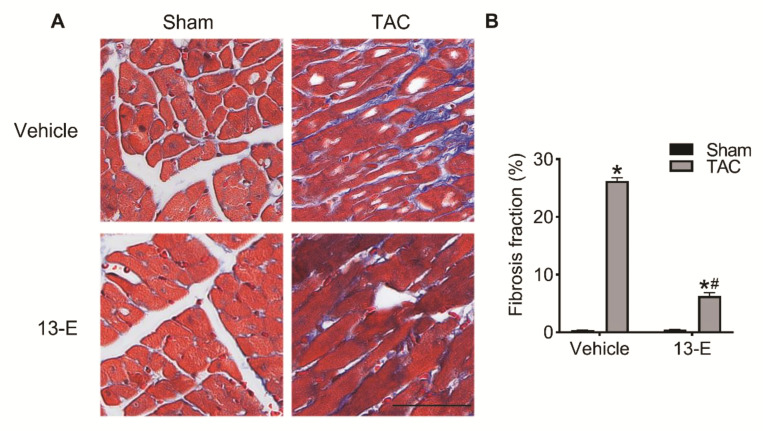
Eight-week-old mice were treated with vehicle or 20 mg/kg **13-E** (i.p.), which were performed with sham or TAC operations for four weeks (*n* = 3 for each group): (**A**) Histological sections of mice hearts were stained with Masson trichrome (Scale Bars: 50 μm); (**B**) Quantification of cardiac fibrosis fraction. The results are presented as mean ± SEM. * *p* < 0.05, versus sham; # *p* < 0.05, versus vehicle + TAC. Comparisons across all groups were performed by two-way ANOVA with Tukey’s post hoc multiple comparisons.

Interstitial fibrosis is a distinguishing feature of cardiac hypertrophy; as a consequence, we analyzed the extent of myocardial fibrosis in the mice. Masson trichrome staining was used to evaluate collagen deposition. The collagen fibers stain blue, the muscles stain red and the nuclei stain black. As expected, TAC surgery resulted in cardiac hypertrophy, reflected in the deposition of myocardial interstitial collagen. The administration of **13-E** significantly decreased the fibrosis fraction in TAC mice compared to the vehicle group (*n* = 3 for each group) (Figure 6). These results indicate that **13-E** plays a vital role in preventing TAC-induced cardiac fibrosis. In summary, **13-E** exhibits an obvious cardioprotective effect in In Vivo pressure overload-induced cardiac hypertrophy murine models by reducing myocardial interstitial fibrosis.

### 2.7. **13-E** Regulates the AMPK Signaling Pathway and Influences Fatty Acid Metabolic Processes

**Figure 7:**
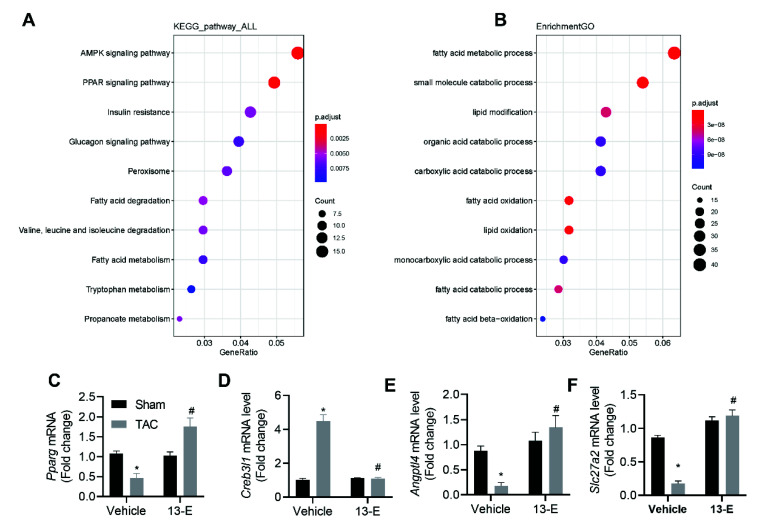
(**A**) KEGG pathway analysis and (**B**) GO analysis of different genes between TAC and TAC+**13-E** treatment groups. (**C**–**F**) mRNA values of *Pparg*, *Creb3l1*, *Angptl4* and *Slc27a2* were analyzed in vehicle or **13-E** treatments in sham- or TAC-operated mice. The results are presented as mean ± SEM. * *p* < 0.05, versus sham; # *p* < 0.05, versus vehicle + TAC. Comparisons across all groups were performed by two-way ANOVA with Tukey′s post hoc multiple comparisons.

In order to identify candidate genes and pathways that mediate the **13-E**-induced protection against cardiac hypertrophy, we performed an unbiased transcriptomic analysis on the left ventricular tissue of mice 4 weeks following the TAC and **13-E** treatments. Using RNA sequencing (RNA-seq) analysis, we identified 764 differentially expressed genes (*p* adjust < 0.05), in which 356/764 (47%) were downregulated by TAC, whereas only 408/764 (53%) were upregulated (Table S1). We then performed Kyoto Encyclopedia of Genes and Genomes (KEGG) pathway analysis, and the results revealed that several biological processes were altered by the **13-E** treatment, including the AMPK signaling pathway, the PPAR signaling pathway and the insulin resistance and glucagon signaling pathway (Figure 7A). The majority of the genes were clustered in the AMPK pathway, which is an interesting observation that we speculated may reflect a regulation of cellular metabolism induced by **13-E**. Gene Ontology (GO) enrichment analysis confirmed this speculation that the majority of the genes were clustered in fatty acid metabolic processes (Figure 7B).

We then verified the expression of several genes in fatty acid metabolic processes using Q-PCR (Figure 7C–F). *Pparg* encodes peroxisome proliferator-activated receptor gamma (PPARγ), which plays key roles in the storage and mobilization of lipids and in glucose metabolism [24]. The level of *Pparg* reduced significantly in TAC mice, and **13-E** treatment restored its level. cAMP responsive element-binding protein 3-like 1 (CREB3L1) is a member of the CREB3 family of transcription factors. They regulate the expression of a large variety of genes and play roles in ER stress and lipid metabolism [25]. The level of *Creb3l1* increased significantly in TAC mice, which may indicate that enhanced ER stress and **13-E** treatment reduced its level. Accumulating evidence indicates that Angiopoietin-like 4 (ANGPTL4) is associated with the risk of atherosclerosis and type 2 diabetes [26]. SLC27A2 is a member of solute carrier family 27, and it enables fatty acid transmembrane transporters [27]. We found that the level of *Angptl4* and *slc27a2* decreased significantly in the heart tissue of TAC mice, and **13-E** treatment reduced the elevations.

## 3. Discussion and Conclusions

Cardiac hypertrophy is a major health problem worldwide, and it is a complex process driven by simultaneous changes in hemodynamics, such as hypertension, characterized by increased heart mass. Pathological cardiac hypertrophy is a key risk factor for heart failure, with systolic and diastolic dysfunction and impaired cardiac function. Clinical drugs that can be used to treat cardiac hypertrophy are limited, largely because of the complex etiology and multiple risk factors during the development of cardiac hypertrophy. Therefore, a multi-target therapeutic strategy may be a better choice for the treatment of cardiac hypertrophy [28]. Polyphenolic isochroman-4-one natural product XJP showed multiple cardioprotective effects, including anti-hypertension, anti-atherosclerosis and anti-inflammation, in our previous work. The current study was performed to determine whether the hydrogen-sulfide-releasing isochroman-4-one hybrid has any therapeutic potential in alleviating cardiac hypertrophy.

Results from this work indicate that hydrogen-sulfide-releasing isochroman-4-one hybrid **13-E** prevents PE-induced cardiac hypertrophy in isolated neonatal rat ventricular cardiomyocytes effectively, whereas the components of **13-E**, XJP and H_2_S-releasing donor TBZ only attenuate PE-induced cardiac hypertrophy slightly. These results confirm that compound **13-E** exhibits its cardioprotective effects as a whole. Importantly, the development of cardiac hypertrophy induced by TAC can be efficiently alleviated by compound **13-E** In Vivo. In previous work, XJP has been shown to specifically reduce LPS-accelerated vascular inflammation as a novel ACEI [7]. ACEIs have been reported to be effective in reducing left ventricular mass in hypertension and heart failure [29]. These studies are in accordance with what was observed in this study, i.e., that **13-E** can efficiently reduce HW/BW and reduce the thickness of the left ventricular posterior wall, LVID and IVS. However, according to our previous reports, XJP can reduce blood pressure effectively in spontaneously hypertensive rats. These anti-hypertrophic effects seem to be derived from a direct protective effect on cardiomyocytes, rather than as a consequence of reductions in blood pressure, as compound **13-E** can alleviate hypertrophy in cultured cardiomyocytes. Interstitial fibrosis is an important marker of cardiac dysfunction and a predictor for the poor prognosis of cardiac hypertrophy. In animals treated with compound **13-E**, a reduction in interstitial fibrosis determined by Masson staining was observed, indicating that compound **13-E** can improve cardiac compliance. As a result, cardiac functions including ejection fraction and fractional shortening were improved in compound **13-E**-treated animals.

ATP synthesis and catabolism are dynamic processes in the maintenance of cellular homeostasis, especially in cardiomyocytes with high energy remanding. It is well-known that cellular energy depletion can activate AMPK by increasing the ratio of AMP/ATP. In normal hearts, fatty acid oxidation is the main source of energy for cardiomyocyte constriction. However, during cardiac hypertrophy, fatty acid oxidation is impaired, and there is an imbalance between energy production and consumption, which can incite many signaling pathways associated with cellular energetic metabolism. In fact, H_2_S has been reported to activate AMPK during cardiac dysfunction, and interestingly, we also found that **13-E** mainly influences genes associated with fatty acid oxidation and the AMPK pathway, indicating that the protection effects of **13-E** on cardiac hypertrophy may be mediated by regulating cellular energetic metabolism.

The combination principle is commonly used to design H_2_S-releasing drugs, as these hybrids often possess superior pharmacodynamic and pharmacokinetic characteristics. The current study demonstrates that hybrid **13-E** effectively reduces cardiac hypertrophy and fibrosis and improves cardiac function in TAC-induced cardiac hypertrophy. Interestingly, the co-administration of purified XJP combined with TBZ was also found to alleviate hypertrophy; however, this drug combination is less efficient than hybrid **13-E**, suggesting the independence of compound **13-E**. Together with the cardioprotective effects of XJP and H_2_S, compound **13-E** can be developed to be a promising drug candidate for the treatment of cardiac hypertrophy in the future.

## 4. Experimental Section

### 4.1. Chemistry

^1^H NMR and ^13^C NMR spectra were recorded on a Bruker AV-300 NMR, the deuterated solvents were CDCl_3_ and DMSO-*d*_6_ and the mass spectra were obtained on an Agilent 1100-LC-MSD-Traps/SL. All reagents and solvents were commercially available and were used without further purification. Silicagel 60 H (200–300 mesh), manufactured by Qingdao Haiyang Chemical Group Co., Ltd. (Qingdao, China), was used for general chromatography.

#### 4.1.1. 6-(Benzyloxy)-7-methoxy-3-methylisochroman-4-one (**4**)

Compound **1** (5.0 g, 1 eq) was added to the appropriate amount of methanol solution, and NaBH_4_ (0.4 g, 1 eq) was added several times. After stirring for 30 min at room temperature, the reaction solution became clear. After the reaction was completed, the reaction solution was concentrated under reduced pressure, and a small amount of water was slowly added. The white solid was separated out, and about 6.2 g of compound **2** was obtained by a brewer funnel and was used directly without further purification.

Compound **2** (6.0 g, 1 eq) was dissolved in DMF, *N*-methoxy-*N*-methyl-2-bromopropanamide (2.3 mL, 1.5 eq), and a catalytic amount of NaH was added. The reaction was stirred at R.T. for 30 min. After the reaction was completed, the reaction solution was transferred to the separation funnel, and ethyl acetate was extracted three times. Then, the organic layer was combined, was washed with saturated NaCl solution, was dried to obtain about 5.0 g of compound **3** and was used directly without further purification.

Compound **3** (5.0 g, 1 eq) was placed in a three-neck flask and was dissolved in anhydrous THF in a nitrogen-protected atmosphere. Under −78 °C, the *tert*-butyl lithium solution (25 mL, 2.2 eq) was injected into the reaction bottle and was stirred for about 15 min. After the reaction was completed, the ammonium chloride solution was added to quench the reaction. The excess THF was first spun out, and then it was extracted with water and ethyl acetate three times. About 2.5 g of white solid compound **4** was obtained through column purification (petroleum ether: ethyl acetate 10:1). ^1^H NMR (300 MHz, CDCl_3_) *δ* 7.55 (s, 1H), 7.47–7.26 (m, 5H), 6.61 (s, 1H), 5.16 (s, 2H), 4.84 (s, 2H), 4.19 (q, *J* = 6.6 Hz, 1H), 3.92 (s, 3H), 1.49 (d, *J* = 6.6 Hz, 3H); ^13^C NMR (75 MHz, CDCl3) *δ* 194.77, 154.57, 147.81, 137.23, 136.33, 128.64, 128.11,127.55, 122.33, 110.02, 106.18, 77.98, 70.84, 66.60, 56.22, 15.88; HRMS (ESI) calculated for C_18_H_18_O_4_ [M + Na]^+^ 321.1097, found 321.1110. White solid; yield: 2.5 g, 93%.

#### 4.1.2. 6-Hydroxy-7-methoxy-3-methylisochroman-4-one (**5**)

Compound **4** was dissolved in methanol, and 10% Pd/C was added. Hydrogen was completely replaced, and the reaction was carried out at room temperature overnight. Pd/C was removed by filtration, and the solvent was dried and passed through the column (petroleum ether: ethyl acetate 2:1) to obtain about 1.0 g of white solid compound **5**. ^1^H NMR (400 MHz, CDCl_3_) *δ* 7.53 (s, 1H), 6.59 (s, 1H), 5.82 (s, 1H), 4.83 (d, *J* = 4.3 Hz, 2H), 4.20 (q, *J* = 6.6 Hz, 1H), 3.94 (s, 3H), 1.49 (d, *J* = 6.7 Hz, 3H). White solid; yield: 1.0 g, 70%.

#### 4.1.3. 6-((6-Bromohexyl)oxy)-7-methoxy-3-methylisochroman-4-one (**6**)

K_2_CO_3_ (276 mg, 2.0 eq) was added to a solution of compound **5** (100 mg, 1 eq) in anhydrous acetone, and the mixture was refluxed for 30 min. Then, 1, 6-dibromohexane (210 μL, 3 eq) was added and the mixture and was refluxed for 2 h. After being filtrated and concentrated under reduced pressure, followed by being purified by flash column chromatography with n-hexane/ethyl acetate (8:1, *v*/*v*) as an eluent, compound **6** was afforded as a white solid in a yield of 85%. ^1^H NMR (400 MHz, CDCl_3_) δ 7.46 (s, 1H), 6.60 (s, 1H), 4.91–4.80 (m, 2H), 4.22 (dd, *J* = 6.7, 1.0 Hz, 1H), 4.09–4.02 (m, 2H), 3.92 (s, 3H), 3.41 (t, *J* = 6.8 Hz, 2H), 1.88 (dd, *J* = 6.9, 3.4 Hz, 4H), 1.50 (d, *J* = 6.7 Hz, 7H).

#### 4.1.4. 4-((6-((7-Methoxy-3-methyl-4-oxoisochroman-6-yl)oxy)hexyl)oxy)benzonitrile (**7**)

4-Cyanophenol (40 mg, 1.2 eq) was dissolved in anhydrous acetonitrile, and K_2_CO_3_ (130 mg, 3 eq) was added. After refluxing for 30 min in a nitrogen-protected atmosphere, compound **6** (100 mg, 1 eq) was added. The whole reaction system continued to reflux for 6 h. After being filtrated and concentrated under reduced pressure, the filtrate was diluted with ethyl acetate, was washed with water and brine and was dried with anhydrous Na_2_SO_4_. Purified by flash column chromatography, compound **7** (about 100 mg) was afforded as a white solid in a yield of 76%. ^1^H NMR (400 MHz, CDCl_3_) *δ* 7.59–7.55 (m, 2H), 7.47 (s, 1H), 6.96–6.90 (m, 2H), 6.60 (s, 1H), 4.92–4.81 (m, 2H), 4.22 (dd, *J* = 6.7, 1.0 Hz, 1H), 4.07 (t, *J* = 6.8 Hz, 2H), 4.01 (t, *J* = 6.4 Hz, 2H), 3.92 (s, 3H), 1.95–1.79 (m, 4H), 1.55 (s, 4H), 1.51 (d, *J* = 6.7 Hz, 3H).

#### 4.1.5. 4-((6-((7-Methoxy-3-methyl-4-oxoisochroman-6-yl)oxy)hexyl)oxy)benzothioamide (**13-E**)

Compound **7** (100 mg, 1 eq) was dissolved in DMF, and NaHS (50 mg, 2 eq) and MgCl_2_·6H_2_O (60 mg, 1 eq) were added. After stirring for 60 min at room temperature and being filtrated and concentrated under reduced pressure, followed by being purified by flash column chromatography, compound **13-E** was afforded as a yellow solid in a yield of 60%, and the mass was about 79 mg. ^1^H NMR (300 MHz, DMSO-d_6_) *δ* 9.65 (s, 1H), 9.32 (s, 1H),7.94 (d, J = 8.55 Hz, 2H), 7.32 (s, 1H), 6.94 (m, 3H), 4.84 (s, 2H), 4.27 (q, *J* = 6.75 Hz, 1H), 4.01 (m, 4H), 3.85 (s, 3H), 1.75 (s, 4H), 1.48 (s, 4H), 1.34 (d, *J* = 6.60 Hz, 3H); ^13^C NMR (75 MHz, CDCl_3_) δ 203.2, 199.1, 166.0, 158.6, 152.2, 142.0, 135.8, 134.1, 126.2, 118.1, 112.9, 111.8, 81.6, 72.8, 72.3, 70.4, 60.6, 33.2, 29.9, 29.9, 20.4; HRMS (ESI) calcd for C_24_H_29_NO_5_S [M + H]^+^ 444.1839, found 444.1847; HPLC retention time: 4.676 min. HPLC purity: 98.5%. Yellow solid; yield: 79 mg, 85%; Melting point: 133–135 °C.

### 4.2. H_2_S Release Experiment

Sodium phosphate buffer was used to prepare the stock solution of Na_2_S (20 mM) in a 100 mL volumetric flask. Aliquots of Na_2_S stock solution were transferred into a 50 mL volumetric flask to obtain standard solutions of 5, 10, 20, 40, 60, 80, 100 and 150 mM, respectively. An amount of 1 mL of each standard solution was added to react with the MB cocktail (200 mL of 30 mM FeCl_3_ in 1.2 M HCl, 200 mL of 20 mM *N*, *N*-dimethyl-1,4-phenylenediaminesulfate in 7.2 M HCl and 100 mL of 1% *w*/*v* of Zn(OAc)_2_ in H_2_O) at room temperature for 20 min in a triplicate manner. The mixture was measured at 670 nm in a UV-Vis spectrophotometer, and then the Na_2_S calibration curve was obtained. In order to promote the compounds to release H_2_S, L-cysteine was used as an accelerator. All compounds were dissolved in THF solution (40 mM) and were added into a phosphate buffer in the presence of L-cysteine (1 mM). Then, 2 mL of mixture was transferred to a colorimetric cuvette containing MB^+^ cocktail in the designated time. After incubation for 20 min, the absorbance of each compound was analyzed by a UV-Vis spectrophotometer at 670 nm. The H_2_S concentration of each derivative was calculated through a standard curve.

### 4.3. Determination of Cytotoxicity

Cytotoxicity was evaluated with the colorimetric MTT [3-(4,5-dimethyl-2-thiazolyl)-2,5-diphenyl-2H-tetrazolium bromide] assay. CHO cells were seeded at 5 × 10^4^ cells/well in 96-well plates. After 24 h, the medium was removed and replaced with the tested compounds at different concentrations for 24 h at 37 °C. After the replacement of the tested compounds with 80 μL of medium and 20 μL of MTT in PBS (0.5 mg/mL, final concentration), the cells were incubated for another 4 h. After the removal of MTT, the formazan crystals were dissolved in DMSO. The amount of formazan was measured (570 nm). Cell viability was expressed as the percentage of control cells and was calculated using the formula Ft/Fnt × 100, where Ft is the absorbance of the treated neurons after subtracting the absorbance of the zero-day control, and Fnt is the absorbance of the untreated neurons after subtracting the absorbance of the zero-day control.

### 4.4. Animals

All animal care and experiments were approved by the Committee on Animal Care and Use of Nanjing Medical University and were conducted in compliance with the Guide for the Care and Use of Laboratory Animals published by the U.S. National Institutes of Health. All mice were housed in a controlled environment, with regulations of temperature (22 ± 1 °C) and humidity (55%), a 12:12 h dark–light cycle and being fed with a standard chow diet. A total of 32 male C57BL/6 mice at the age of 8 weeks were randomly divided into four groups: vehicle + sham (*n* = 8), **13-E** + sham (*n* = 8), vehicle + TAC (*n* = 8) and **13-E** + TAC (*n* = 8). All TAC group mice were subjected to minimally invasive TAC surgery. Briefly, mice were anesthetized with 5% isoflurane, were maintained with 2.5% isoflurane during surgery and were secured to an operating table. The hair on the neck and chest was removed using a depilatory agent, and then the surgery area was disinfected with alcohol. The chest was opened, and the aortic arch was isolated by blunt dissection through an intercostal incision. The transverse aorta was constricted by a 7-0 silk suture ligature tied firmly against a 27-gauge needle, and then the needle was subsequently removed after ligation. The sham mice were subjected to the same surgical procedure without constriction of the aorta. The incision was then closed. After surgery, the mice were allowed to fully recover on a heating pad and were housed in standard housing conditions.

### 4.5. Neonatal Rat Cardiomyocyte Isolation, Culture and Treatment

Cardiomyocytes were isolated from neonatal rats of 1 to 4 days old. After disinfecting the chest with 75% ethanol, the sternums were cut, and the hearts were removed. The residual blood clots and atria were removed in Dulbecco’s Modified Eagle Medium (DMEM), and the ventricular tissues were minced by dissecting scissors. The ventricular tissues were digested by mixed enzyme solution (0.25% Trypsin-EDTA) in a 37 °C water bath for 5 min. The supernatant was collected and neutralized with DMEM medium containing 20% fetal bovine serum (FBS). Repeated digestions were performed 7–9 times until cells were isolated completely. The collected cell suspension was centrifuged at 2000 rpm for 10 min. The sediments were resuspended in DMEM supplemented with 10% FBS. The cells were then plated with different culture dishes according to the specific experimental requirements at 37 °C in the presence of 5% CO_2_ in a humidified incubator. After culturing in a serum-free medium for 6–8 h, the primary cardiomyocytes were incubated for 48 h with 50 µmol/L of phenylephrine (PE) to induce cardiomyocyte hypertrophy In Vitro, and phosphate buffer saline (PBS) was used as a control under the conditions of 37 °C, 5% CO_2_ and 95% O_2_.

### 4.6. Echocardiography

Cardiac functions were assessed by echocardiography with Vevo 770 after TAC or sham surgery for 4 weeks. Briefly, mice were anesthetized with 2% isoflurane and were adjusted to maintain heart rates in the range of 415–460/min during echocardiogram acquisition. Transthoracic echocardiography of the left ventricle was performed to measure LV wall thickness, LV chamber size, LV function and LV mass. The parameters of cardiac function that were collected include: LVEF, LVFS, left ventricular volume (LV), LVPW, LVID and IVS.

### 4.7. Histological Staining

After surgery, the heart tissues were fixed with 4% polyformaldehyde and were finally embedded into paraffin. The heart-embedded paraffin blocks were cut into 5 µm sections using a microtome and were mounted on slides. To evaluate cardiac histological changes and fibrosis, the paraffin-embedded heart sections were dewaxed, rehydrated and subjected to H and E staining and Masson’s trichrome. Images were taken by a light microscope. The images were analyzed by using the ImageJ analysis system.

## Data Availability

The data presented in this study are available within the article and Supplementary Materials.

## Supplementary Materials

The spectra of ^1^H-NMR and ^13^C-NMR for the target compound are available online: https://www.mdpi.com/article/10.3390/molecules27134114/s1, Figure S1: 8 weeks old mice were treated with Vehicle or **13-E**, which were performed with Sham or transverse aortic constriction (TAC) operation for 4 weeks (*n* = 8 for each group). Heart rates were detected for each group. The results were presented as mean ± SEM. ns: no significant; Figure S2: 8 weeks old mice were treated with Vehicle or **13-E**, which were performed with Sham or TAC operation for 4 weeks (*n* = 8 for each group). (A,B) Left ventricular diastolic and systolic volume (LV vol:d; LV vol:s; ul). (C,D) diastolic and systolic interventricular septum thickness (IVS:d; IVS:s; mm). (E) Left ventricular posterior wall diastolic dimension (LVPW; d, mm) and (F) left ventricular diastolic internal dimension (LVID; d, mm). The results were presented as mean ± SEM. * *p* < 0.05, versus Sham; # *p* < 0.05, versus Vehicle + TAC. Comparisons across all groups were performed by Two-way ANOVA with Turkey′s post hoc multiple comparisons; Table S1. DEG_TAC_vs_13E_fc_1.5_p_0.05.

## Sample Availability

Samples of the compounds are not available from the authors.

## References

[1] Nakamura M., Sadoshima J. (2018). Mechanisms of physiological and pathological cardiac hypertrophy. Nat. Rev. Cardiol..

[2] Shimizu I., Minamino T. (2016). Physiological and pathological cardiac hypertrophy. J. Mol. Cell. Cardiol..

[3] Lei H., Hu J., Sun K., Xu D. (2021). The role and molecular mechanism of epigenetics in cardiac hypertrophy. Heart Fail. Rev..

[4] Tham Y.K., Bernardo B.C., Ooi J.Y., Weeks K.L., McMullen J.R. (2015). Pathophysiology of cardiac hypertrophy and heart failure: Signaling pathways and novel therapeutic targets. Arch. Toxicol..

[5] Wu X., Liu Z., Yu X.Y., Xu S., Luo J. (2021). Autophagy and cardiac diseases: Therapeutic potential of natural products. Med. Res. Rev..

[6] Khan J., Deb P.K., Priya S., Medina K.D., Devi R., Walode S.G., Rudrapal M. (2021). Dietary flavonoids: Cardioprotective potential with antioxidant effects and their pharmacokinetic, toxicological and therapeutic concerns. Molecules.

[7] Fu R., Chen Z., Wang Q., Guo Q., Xu J., Wu X. (2011). XJP-1, a novel ACEI, with anti-inflammatory properties in HUVECs. Atherosclerosis.

[8] Bai R., Liu J., Zhu Y., Yang X., Yang C., Kong L., Wang X., Zhang H., Yao H., Shen M. (2012). Chiral separation, configurational identification and antihypertensive evaluation of (±)-7,8-dihydroxy-3-methyl-isochromanone-4. Bioorg. Med. Chem. Lett..

[9] Xie S., Li X., Yu H., Zhang P., Wang J., Wang C., Xu S., Wu Z., Liu J., Zhu Z. (2019). Design, synthesis and biological evaluation of isochroman-4-one hybrids bearing piperazine moiety as antihypertensive agent candidates. Bioorg. Med. Chem..

[10] Fu R., Wang Q., Guo Q., Xu J., Wu X. (2013). XJP-1 protects endothelial cells from oxidized low-density lipoprotein-induced apoptosis by inhibiting NADPH oxidase subunit expression and modulating the PI3K/Akt/eNOS pathway. Vasc. Pharmacol..

[11] Uras G., Manca A., Zhang P., Markus Z., Mack N., Allen S., Bo M., Xu S., Xu J., Georgiou M. (2021). In Vivo evaluation of a newly synthesized acetylcholinesterase inhibitor in a transgenic *Drosophila* model of Alzheimer’s disease. Front. Neurosci..

[12] Abe K., Kimura H. (1996). The possible role of hydrogen sulfide as an endogenous neuromodulator. J. Neurosci..

[13] Wang R. (2002). Two’s company, three’s a crowd: Can H_2_S be the third endogenous gaseous transmitter?. FASEB J..

[14] Murphy B., Bhattacharya R., Mukherjee P. (2019). Hydrogen sulfide signaling in mitochondria and disease. FASEB J..

[15] Citi V., Piragine E., Testai L., Breschi M.C., Calderone V., Martelli A. (2018). The role of hydrogen sulfide and H_2_S-donors in myocardial protection against ischemia/reperfusion injury. Curr. Med. Chem..

[16] Xie L., Gu Y., Wen M., Zhao S., Wang W., Ma Y., Meng G., Han Y., Wang Y., Liu G. (2016). Hydrogen sulfide induces Keap1 *S*-sulfhydration and suppresses diabetes-accelerated atherosclerosis via Nrf2 activation. Diabetes.

[17] Meng G., Xiao Y., Ma Y., Tang X., Xie L., Liu J., Gu Y., Yu Y., Park C.M., Xian M. (2016). Hydrogen sulfide regulates krüppel-like factor 5 transcription activity via specificity protein 1 *S*-sulfhydration at Cys664 to prevent myocardial hypertrophy. J. Am. Heart Assoc..

[18] Yao H., Luo S., Liu J., Xie S., Liu Y., Xu J., Zhu Z., Xu S. (2019). Controllable thioester-based hydrogen sulfide slow-releasing donors as cardioprotective agents. Chem. Commun..

[19] Luo S., Gu X., Ma F., Liu C., Shen Y., Ge R., Zhu Y. (2016). ZYZ451 protects cardiomyocytes from hypoxia-induced apoptosis via enhancing MnSOD and STAT3 interaction. Free Radic. Biol. Med..

[20] Luo S., Hieu T.B., Ma F., Yu Y., Cao Z., Wang M., Wu W., Mao Y., Rose P., Law B.Y. (2017). ZYZ-168 alleviates cardiac fibrosis after myocardial infarction through inhibition of ERK1/2-dependent ROCK1 activation. Sci. Rep..

[21] Bai R., Yang X., Zhu Y., Zhou Z., Xie W., Yao H., Jiang J., Liu J., Shen M., Wu X. (2012). Novel nitric oxide-releasing isochroman-4-one derivatives: Synthesis and evaluation of antihypertensive activity. Bioorg. Med. Chem..

[22] Do A.V., Smith R., Tobias P., Carlsen D., Pham E., Bowden N.B., Salem A.K. (2019). Sustained release of hydrogen sulfide (H_2_S) from poly(lactic acid) functionalized 4-hydroxythiobenzamide microparticles to protect against oxidative damage. Ann. Biomed. Eng..

[23] Liu J., Ren H., Xu J., Bai R., Yan Q., Huang W., Wu X., Fu J., Wang Q., Wu Q. (2009). Total synthesis and antihypertensive activity of (+/-)7,8-dihydroxy-3-methyl-isochromanone-4. Bioorg. Med. Chem. Lett..

[24] Janani C., Ranjitha Kumari B.D. (2015). PARP gamma gene—A review. Diabetes Metab. Syndr..

[25] Sampieri L., Giusto P.D., Alvarez C. (2019). CREB3 transcription factors: ER-Golgi stress transducers as Hubs for Cellular Homeostasis. Front. Cell Div. Biol..

[26] Fernández-Hernando C., Suárez Y. (2020). ANGPTL4: A multifunctional protein involved in metabolism and vascular homestasis. Curr. Opin. Hematol..

[27] Khan S., Gaivin R., Abramovich C., Boylan M., Calles J., Schelling J.R. (2020). Fatty acid transport protein-2 regulates glycemic control and diabetic kidney disease progression. JCI Insight.

[28] Wang L., Li Z., Tan Y., Li Q., Yang H., Wang P., Lu J., Liu P. (2018). PARP1 interacts with STAT3 and retains active phosphorylated-STAT3 in nucleus during pathological myocardial hypertrophy. Mol. Cell Endocrinol..

[29] Tamura T., Said S., Harris J., Lu W., Gerdes A.M. (2000). Reverse remodeling of cardiac myocyte hypertrophy in hypertension and failure by targeting of the renin-angiotensin system. Circulation.

