# Supplementary material for: Identification of a Hydrogen-Sulfide-Releasing Isochroman-4-One Hybrid as a Cardioprotective Candidate for the Treatment of Cardiac Hypertrophy
Source: Molecules. 2022 Jun 27;27(13):4114. doi: 10.3390/molecules27134114 (PMC9268299; doi:10.3390/molecules27134114)
Supplement: Supplementary file 1 [file molecules-27-04114-s001.zip › SI/molecules-1737490-supplementary.pdf]

Supporting Information for

Identification of a hydrogen sulfide-releasing isochroman-4-one hybrid as a cardioprotective candidate for the treatment of Cardiac hypertrophy

Yu Wang[a], Yuechen Liu[a], Hongyu Wu[b], Shengtao Xu[b,*], Fenfen Ma[c,d,*]

[a] *Key Laboratory of Cardiovascular and Cerebrovascular Medicine, Nanjing Medical University, Nanjing, PR China*

[b] *State Key Laboratory of Natural Medicines and Department of Medicinal Chemistry, China Pharmaceutical University, 24 Tong Jia Xiang, Nanjing 210009, PR China*

[c] *Department of Pharmacy, Shanghai Pudong Hospital, Fudan Univerisity, Shanghai, 201399, PR China*

[d] *School of Pharmacy, Fudan University, Shanghai 201203, PR China*

Corresponding Authors

*E-mail: mafenfen2005@126.com (F. Ma)

*E-mail: cpuxst@163.com (S. Xu)

Table of Contents

Figure S1 2
Figure S2 3
^{1}H NMR and ^{13}C NMR spectra of intermediates and target compound 13-E. 4
^{1}H NMR of intermediate **5** 4
^{1}H NMR of intermediate **6** 5
^{1}H NMR of intermediate **7** 6
^{1}H NMR and ^{13}C NMR of compound **13-E** 7
HRMS spectrum for compound 13-E 9
HPLC spectrum for compound 13-E 10

Supplementary

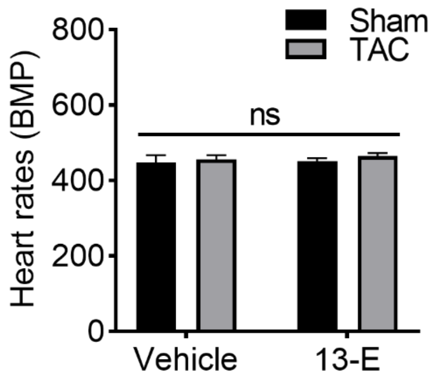

Figure S1. 8 weeks old mice were treated with Vehicle or 13-E, which were performed with Sham or transverse aortic constriction (TAC) operation for 4 weeks (n=8 for each group). Heart rates were detected for each group. The results were presented as mean ± SEM. ns: no significant.

Supplementary

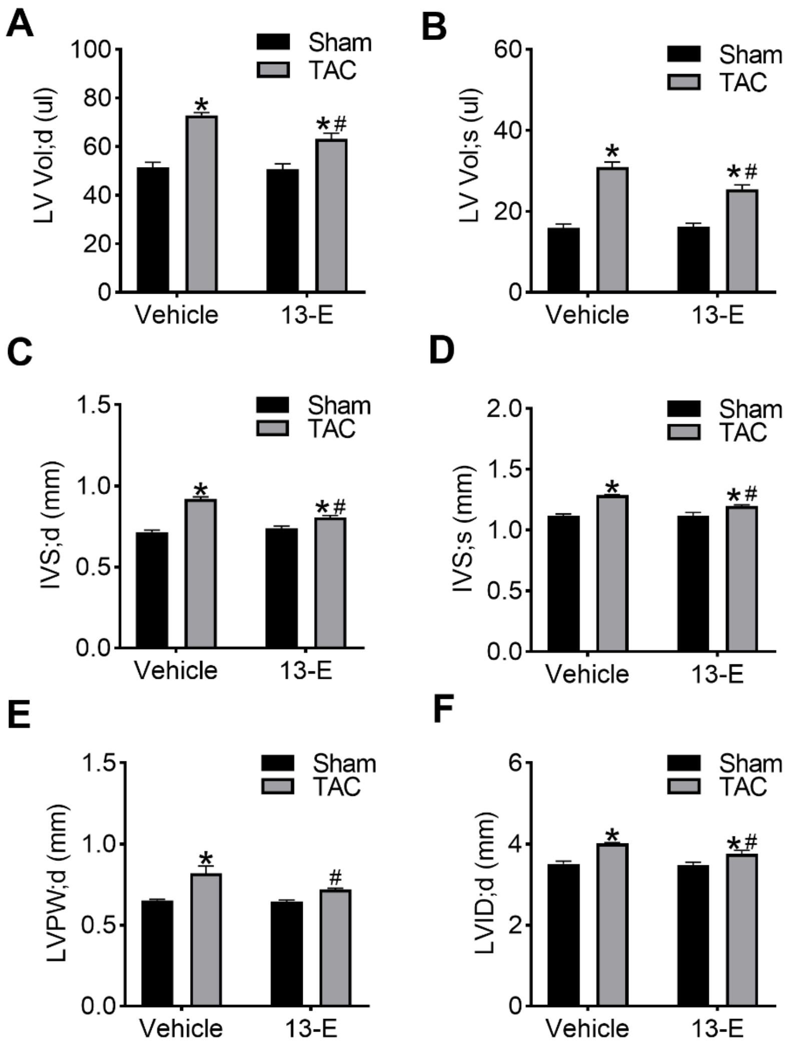

Figure S2. 8 weeks old mice were treated with Vehicle or 13-E, which were performed with Sham or TAC operation for 4 weeks (n=8 for each group). (A-B) Left ventricular diastolic and systolic volume (LV vol:d; LV vol:s; ul). (C-D) diastolic and systolic interventricular septum thickness (IVS:d; IVS:s; mm). (E) Left ventricular posterior wall diastolic dimension (LVPW; d, mm) and (F) left ventricular diastolic internal dimension (LVID; d, mm). The results were presented as mean ± SEM. *$p<0.05$, versus Sham; #$p<0.05$, versus Vehicle + TAC. Comparisons across all groups were performed by Two-way ANOVA with Turkey's post hoc multiple comparisons.

^{1}H NMR and ^{13}C NMR spectra of intermediates and target compound 13-E.

^{1}H NMR of intermediate **5**

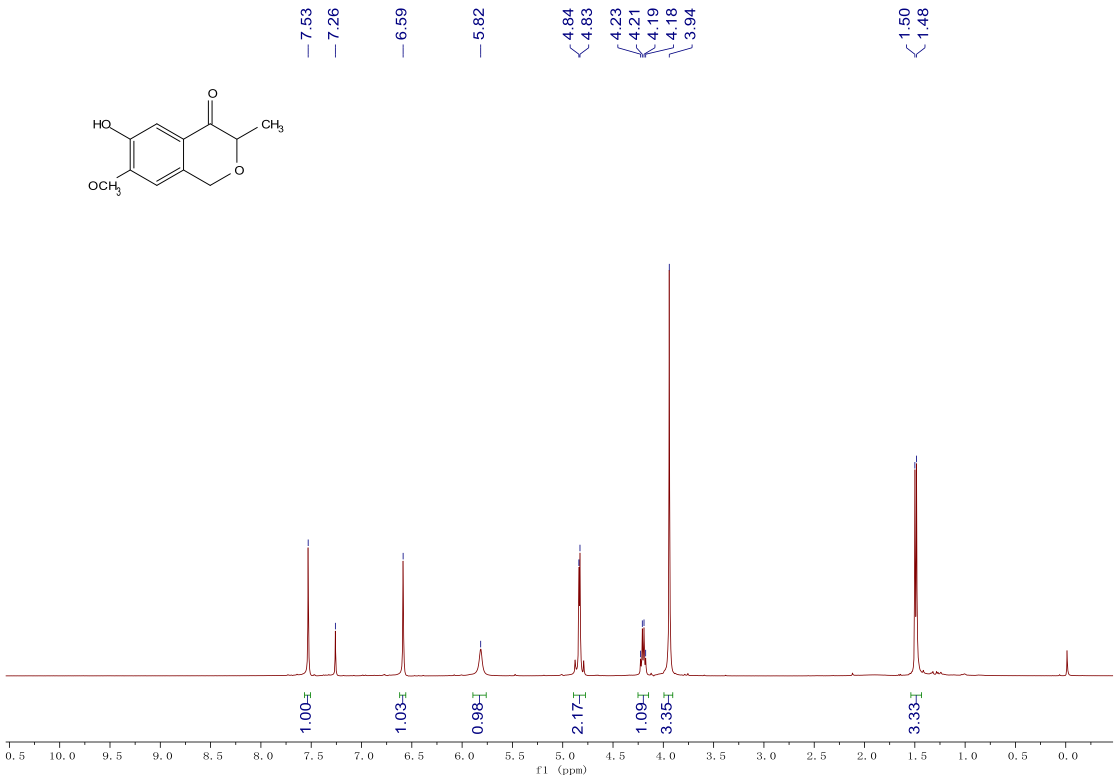

^{1}H NMR (400 MHz, Chloroform-*d*) δ 7.53 (s, 1H), 6.59 (s, 1H), 5.82 (s, 1H), 4.83 (d, J = 4.3 Hz, 2H), 4.20 (q, J = 6.6 Hz, 1H), 3.94 (s, 3H), 1.49 (d, J = 6.7 Hz, 3H).

^{1}H NMR of intermediate **6**

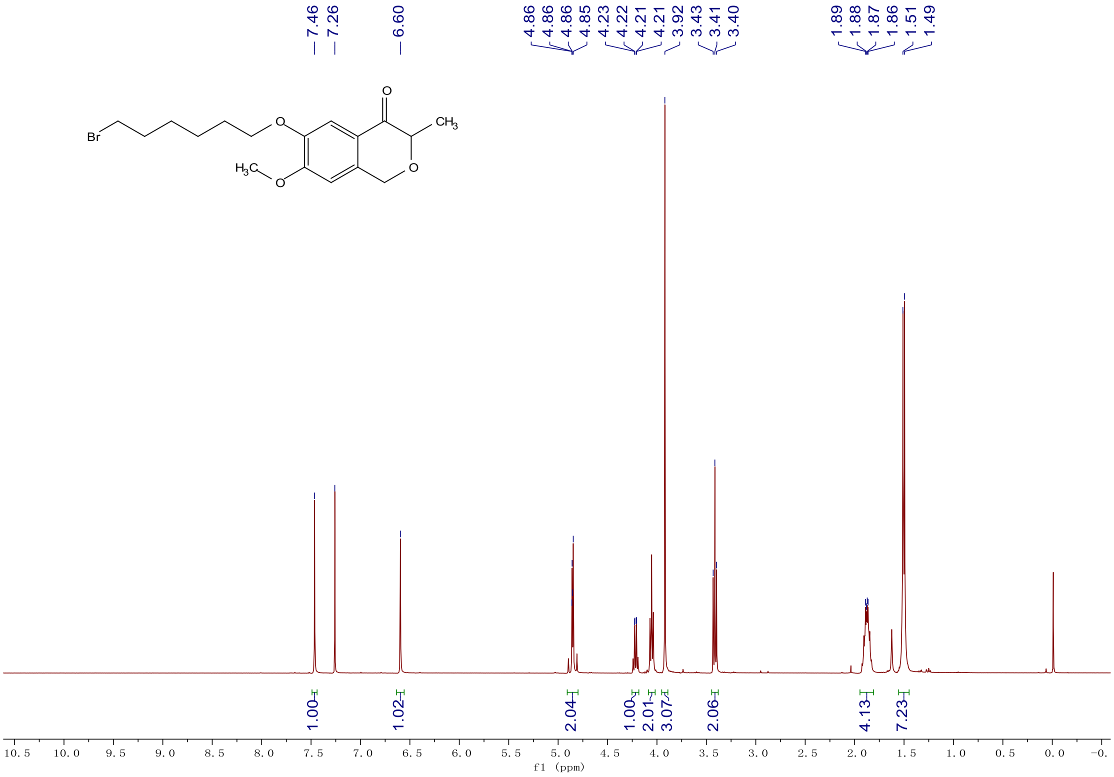

^{1}H NMR (400 MHz, Chloroform-*d*) δ 7.46 (s, 1H), 6.60 (s, 1H), 4.91-4.80 (m, 2H), 4.22 (dd, J = 6.7, 1.0 Hz, 1H), 4.09-4.02 (m, 2H), 3.92 (s, 3H), 3.41 (t, J = 6.8 Hz, 2H), 1.88 (dd, J = 6.9, 3.4 Hz, 4H), 1.50 (d, J = 6.7 Hz, 7H).

^{1}H NMR of intermediate **7**

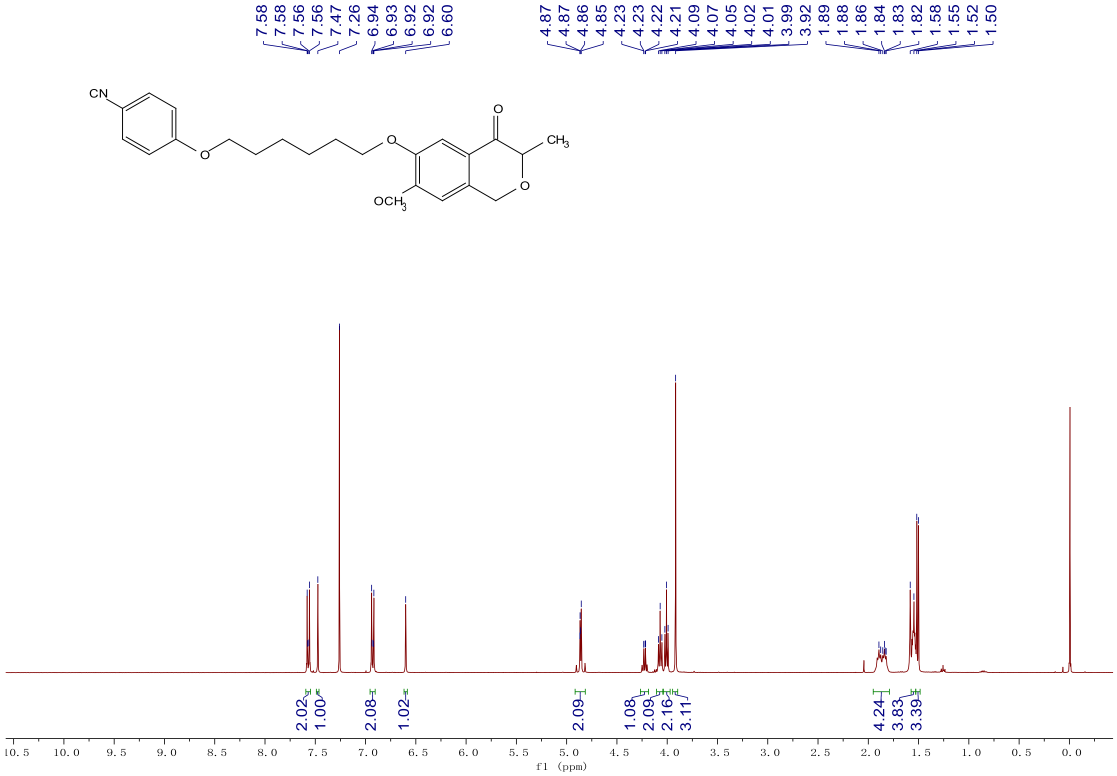

^{1}H NMR (400 MHz, Chloroform-*d*) δ 7.59-7.55 (m, 2H), 7.47 (s, 1H), 6.96-6.90 (m, 2H), 6.60 (s, 1H), 4.92-4.81 (m, 2H), 4.22 (dd, J = 6.7, 1.0 Hz, 1H), 4.07 (t, J = 6.8 Hz, 2H), 4.01 (t, J = 6.4 Hz, 2H), 3.92 (s, 3H), 1.95-1.79 (m, 4H), 1.55 (s, 4H), 1.51 (d, J = 6.7 Hz, 3H).

^{1}H NMR and ^{13}C NMR of compound **13-E**

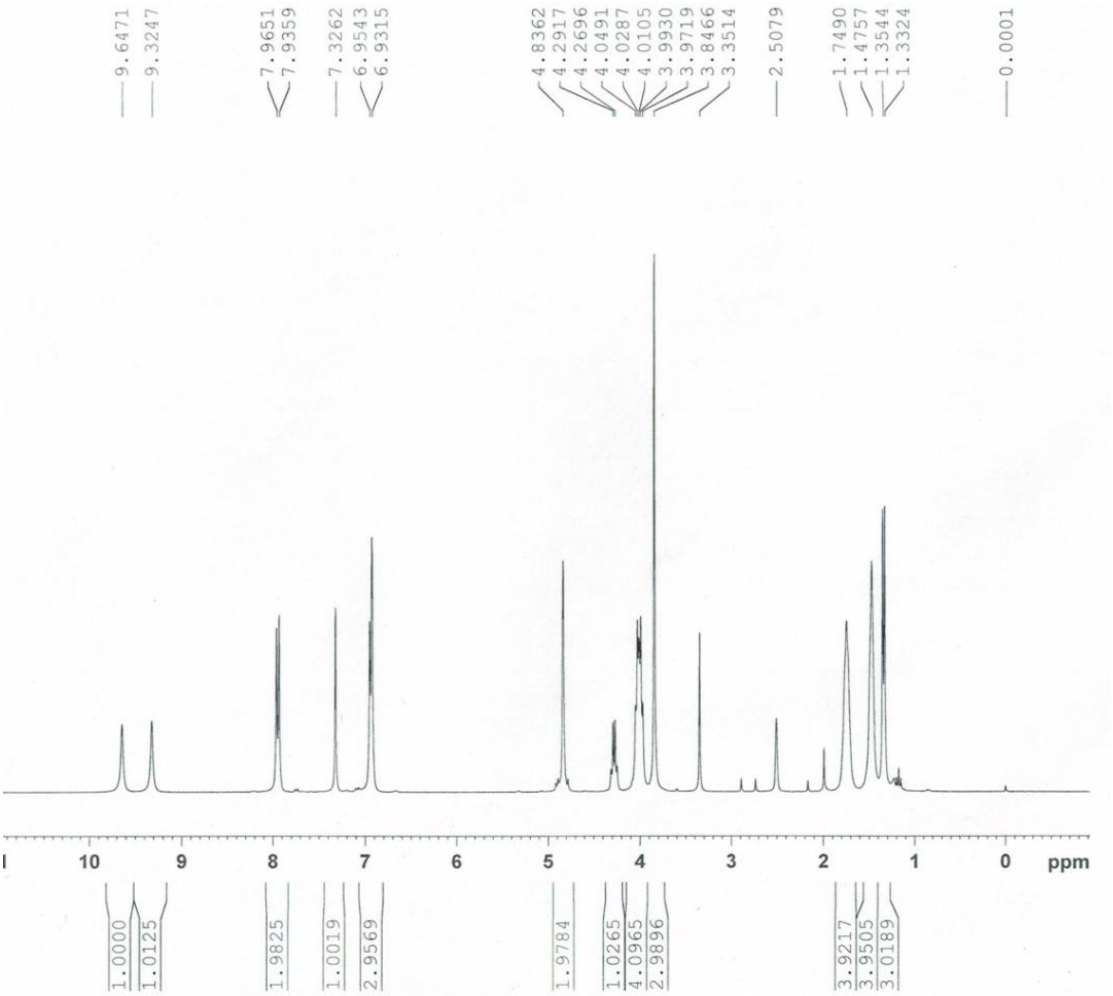

^{1}H NMR (300 MHz, DMSO-d_6) δ 9.65 (s, 1H), 9.32 (s, 1H),7.94 (d, J = 8.55 Hz, 2H), 7.32 (s, 1H), 6.94 (m, 3H), 4.84 (s, 2H), 4.27 (q, J = 6.75 Hz, 1H), 4.01 (m, 4H), 3.85 (s, 3H), 1.75 (s, 4H), 1.48 (s, 4H), 1.34 (d, J = 6.60 Hz, 3H)

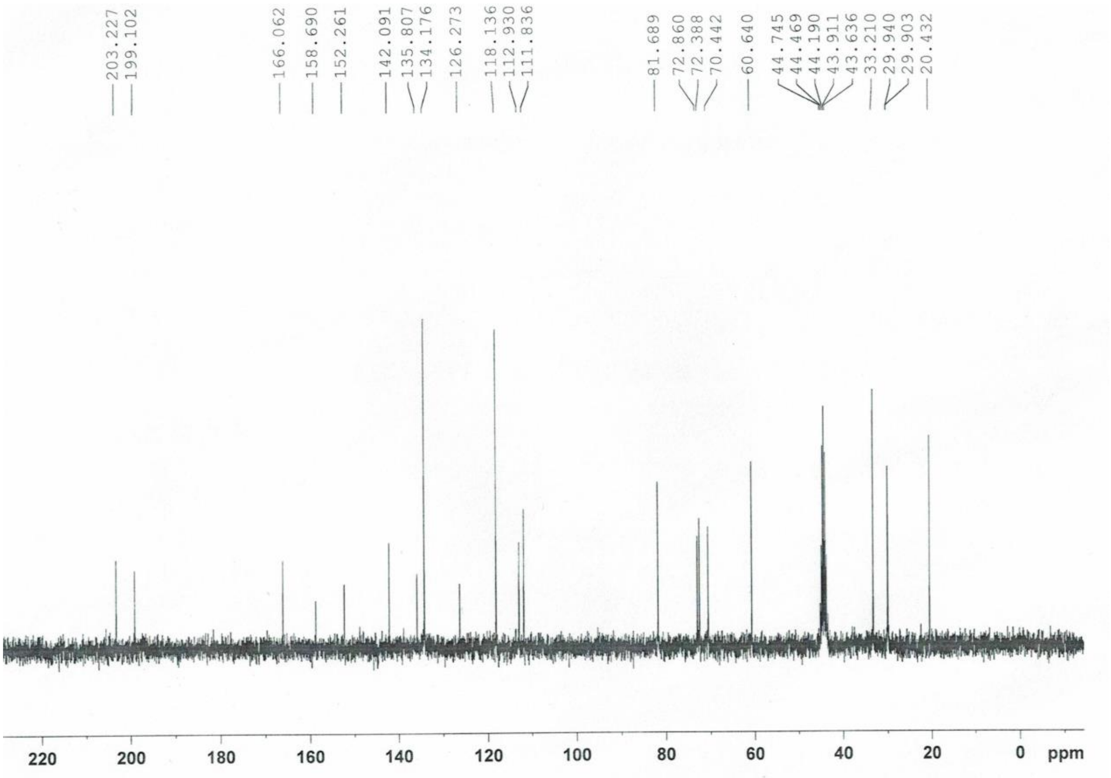

^{13}C NMR (75 MHz, $CDCl_3$) δ 203.2, 199.1, 166.0, 158.7, 152.3, 142.1, 135.8, 134.2, 126.3, 118.1, 112.9, 111.8, 81.7, 72.9, 72.4, 70.4, 60.6, 33.2, 29.94, 29.90, 20.4

HRMS spectrum for compound 13-E

Qualitative Analysis Report

**Data Filename**	POS-HR-9.d	**Sample Name**	POS-HR-9
**Sample Type**	Sample	**Position**	P1-C9
**Instrument Name**	Instrument 1	**User Name**	
**Acq Method**	2MIN-POS.m	**Acquired Time**	4/8/2022 10:27:54 AM
**IRM Calibration Status**	Success	**DA Method**	22222.m
**Comment**			

User Spectra

Fragmentor Voltage	Collision Energy	Ionization Mode
100	0	Esi

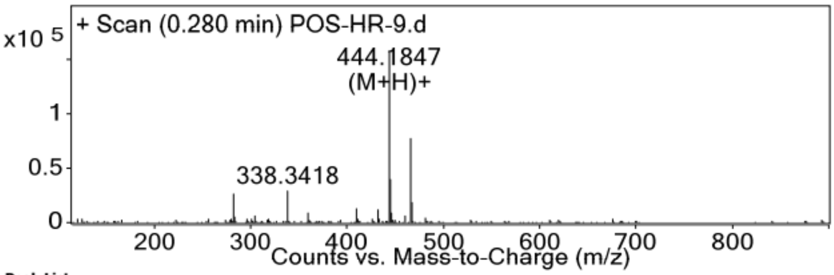

Peak List

m/z	z	Abund	Formula	Ion
282.2788		25854		
338.3418	1	29206		
410.1969		12353		
432.1784		11448		
444.1847	1	156075	C24 H30 N O5 S	(M+H)+
445.1877	1	39318	C24 H30 N O5 S	(M+H)+
466.1668	1	76891		
467.1697	1	17859		
909.3445	1	20902		
910.3458	1	13349		

Formula Calculator Element Limits

Element	Min	Max
C	3	80
H	0	120
O	0	30
N	0	5
S	0	3

Formula Calculator Results

Formula	Best	Mass	Tgt Mass	Diff (ppm)	Ion Species	Score
C18 H27 N4 O9		443.17742	443.1778	0.87	C18 H28 N4 O9	95.36
C24 H29 N O5 S	TRUE	443.17741	443.17664	-1.74	C24 H30 N O5 S	94.95
C25 H25 N5 O S		443.17742	443.17798	1.27	C25 H26 N5 O S	94.4
C20 H29 N O10		443.17741	443.17915	3.91	C20 H30 N O10	89.98
C17 H31 O13		443.17741	443.17647	-2.13	C17 H32 O13	89.57
C22 H27 N4 O4 S		443.17742	443.1753	-4.78	C22 H28 N4 O4 S	87.75
C27 H27 N2 O2 S		443.17741	443.17932	4.31	C27 H28 N2 O2 S	85.6
C18 H35 O8 S2		443.17742	443.17733	-0.18	C18 H36 O8 S2	84.7
C19 H31 N4 O4 S2		443.17742	443.17867	2.82	C19 H32 N4 O4 S2	84.05
C30 H23 N2 O2		443.17741	443.17595	-3.29	C30 H24 N2 O2	82.71

--- End Of Report ---

HPLC spectrum for compound 13-E

〈色谱图〉

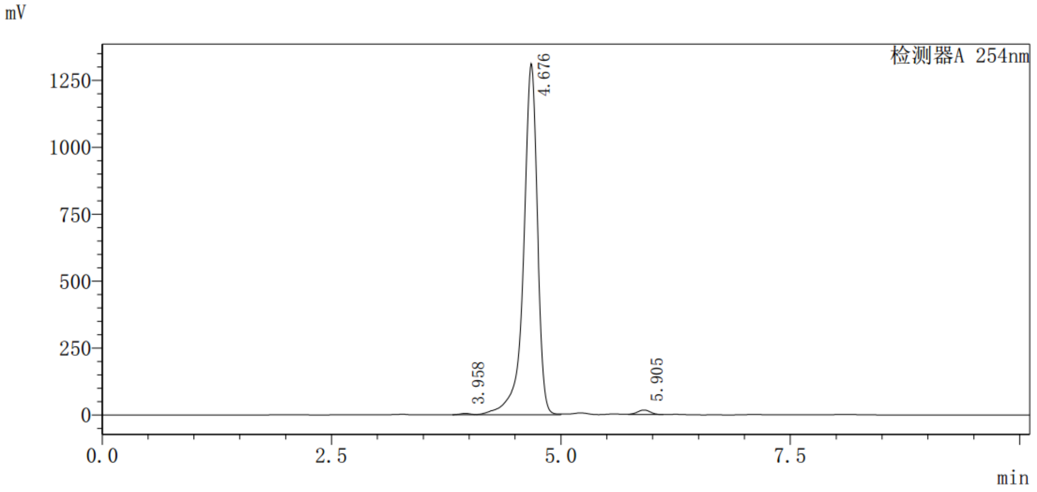

〈峰表〉

检测器A 254nm

峰号	保留时间	面积	高度	浓度	浓度单位	标记	化合物名
1	3.958	35271	4671	0.252			
2	4.676	13791902	1311418	98.524		V	
3	5.905	171279	17140	1.224			
总计		13998452	1333229				

No.	Retention time (min)	Area	Area%
1	3.958	35271	0.252
2	4.676	13791902	98.524
3	5.905	171279	1.224
Total		13998452	100.0
